# Draft Genome Sequence of “*Candidatus* Arthromitus” UMNCA01, a Suspected Commensal Isolated from the Gut Microbiome of Commercial Turkey

**DOI:** 10.1128/MRA.01143-19

**Published:** 2020-01-23

**Authors:** Grant A. Hedblom, Kamal Dev, Steven D. Bowden, Bonnie Weber, Sally Noll, David J. Baumler, Timothy J. Johnson

**Affiliations:** a Department of Food Science and Nutrition, University of Minnesota—Twin Cities, St. Paul, Minnesota, USA; b Faculty of Applied Sciences and Biotechnology, Shoolini University, Solan, Himachal Pradesh, India; c Microbial and Plant Genomics Institute, University of Minnesota—Twin Cities, St. Paul, Minnesota, USA; d Biotechnology Institute, University of Minnesota—Twin Cities, St. Paul, Minnesota, USA; e Department of Veterinary and Biomedical Sciences, University of Minnesota—Twin Cities, St. Paul, Minnesota, USA; f Department of Animal Science, University of Minnesota—Twin Cities, St. Paul, Minnesota, USA; University of Maryland School of Medicine

## Abstract

“*Candidatus* Arthromitus” UMNCA01 was recovered from ileal samples of commercial turkey poults and may have probiotic capabilities. The complete genome was determined using the Illumina MiSeq and HiSeq sequencing platforms. The complete genome consists of 1,631,326 bp and has a G+C content of 26.14%, 1,540 coding sequences (CDS), and 37 RNA coding genes.

## ANNOUNCEMENT

A candidate genus of segmented filamentous bacteria, “*Candidatus* Arthromitus” belongs to the family *Clostridiaceae*. These commensal organisms promote adaptive and innate immune responses in murine models in a host-specific manner, prevent diseases, and promote animal growth (1, 2). Different strains of “*Candidatus* Arthromitus” inhabit the ileum region in many vertebrate animals, such as cattle, pigs, chickens, humans, and, as shown more recently, turkeys (3). In an attempt to discern the microbial basis of light turkey syndrome (LTS), a condition where commercial turkey flocks fail to meet their genetic potential weights despite standardized diets and growth conditions (4), Danzeisen et al. performed 16S rRNA sequencing of intestinal microbiome analysis of high-performing and low-performing (based upon flock weights) turkey flocks (5). This analysis revealed that at the age of 2 to 3 weeks, high-performing turkey flocks harbored significantly higher proportions of “*Candidatus* Arthromitus” bacteria than their low-performing counterparts (5). In this study, the genome of a turkey-specific strain of “*Candidatus* Arthromitus” was sequenced from the gut microbiome.

“*Candidatus* Arthromitus” UMNCA01, a Gram-positive bacterium, was recovered from ileal samples harvested from 2-week-old turkey poults from a research turkey flock in barns at the University of Minnesota. The sample was identified for shotgun sequencing by previous 16S rRNA amplicon profiling indicating a high relative abundance of “*Candidatus* Arthromitus” bacteria and light microscopy confirming the presence of high levels of segmented filamentous bacteria. For metagenomic shotgun sequencing, the total genomic DNA was isolated using a Qiagen stool kit (Hilden, Germany). The quantity of the genomic DNA was determined by measuring *A*_260_ using a UV-visible (UV-Vis) spectrophotometer (*A*_260_ = 1 corresponds to 50 ng/μl of double-stranded DNA [dsDNA]). The quality of the genomic DNA was determined by measuring the *A*_260_/*A*_280_ ratio, and a value of 1.8 indicated pure DNA preparation as described (6). Twenty micrograms of metagenomic DNA was used to prepare a paired-end (PE) sequencing library (Nextera XT, Illumina, San Diego, CA), and a PCR amplified library was sequenced using the Illumina MiSeq and HiSeq platforms. The shotgun data were assembled using CLC Genomics Workbench v. 9.0/APRIL-2016, with default parameters, and then contigs were mapped to an existing mouse “*Candidatus* Arthromitus” genome using Mauve (7) to retrieve and arrange “*Candidatus* Arthromitus” sequences (sourced from turkeys) that mapped to those genomes. Following manual curation, unmapped contigs were then filtered from the metagenomic assembly. The final “*Candidatus* Arthromitus” assembly resulted in an average 100× genome coverage with a total number of 1,631,326 bp arranged into 41 contigs. The G+C content of these contigs was 26.14%, with an average contig size of 39,788 bp and an *N*_50_ value of 57,760 bp. The draft genome contains 1,480 protein coding sequences, 37 RNA genes, and 60 pseudogenes. The genome sequence of “*Candidatus* Arthromitus” UMNCA01 was annotated using the National Center for Biological Information (NCBI) Prokaryotic Genome Annotation Pipeline and the best-placed reference protein set of GeneMarkS+ (annotation software v. 4.6) as described (8, 9); the results are summarized in Table 1.

**TABLE 1:** Global statistics of the “*Candidatus* Arthromitus” UMNCA01 genome

Parameter^*a*^	Value
Total sequence length (bp)	1,631,326
No. of genes (total)	1,577
No. of CDS (total)	1,540
No. of genes (coding)	1,480
No. of CDS (coding)	1,480
No. of genes (RNA)	37
No. of rRNAs (16S)	1
No. of partial rRNAs (16S)	1
No. of tRNAs	33
No. of ncRNAs	3
No. of pseudogenes	60
No. of scaffolds	41
Scaffold *N*_50_ (bp)	68,513
Scaffold *L*_50_ (bp)	9
No. of contigs	44
Contig *N*_50_ (bp)	57,760
Contig *L*_50_ (bp)	10

a CDS, coding DNA sequences; ncRNAs, noncoding RNAs.

### Data availability.

This “*Candidatus* Arthromitus” UMNCA01 whole-genome shotgun (WGS) project has the GenBank accession number NZ_LXFF00000000. The version of this project is NZ_LXFF01000000 and consists of sequences LXFF01000001 through LXFF01000041. The filtered assembly and raw sequencing reads can be accessed through BioProject accession number PRJNA319431 and BioSample accession numbers SAMN04889864 and SAMN13392129, respectively.
